# Self-rated global health in the Norwegian general population

**DOI:** 10.1186/s12955-019-1258-y

**Published:** 2019-12-23

**Authors:** Tore Bonsaksen, Øivind Ekeberg, Laila Skogstad, Trond Heir, Tine K. Grimholt, Anners Lerdal, Inger Schou-Bredal

**Affiliations:** 1 Department of Occupational Therapy, Prosthetics and Orthotics, Faculty of Health Sciences, OsloMet – Oslo Metropolitan University, Box 4 St. Olavs Plass, 0130 Oslo, PO Norway; 2 Faculty of Health Studies, VID Specialized University, Sandnes, Norway; 3 Division of Mental Health and Addiction, Oslo University Hospital, Oslo, Norway; 4 Department of Behavioural Sciences in Medicine, University of Oslo, Oslo, Norway; 5 Department of Research, Sunnaas Rehabilitation Hospital, Nesoddtangen, Norway; 6 Norwegian Center for Violence and Traumatic Stress Studies, Oslo, Norway; 7 Institute of Clinical Medicine, University of Oslo, Oslo, Norway; 8 Department of Nursing and Health Promotion, Faculty of Health Sciences, Oslo Metropolitan University, Oslo, Norway; 9 Department for Patient Safety and Research, Lovisenberg Diakonale Hospital, Oslo, Norway; 10 Department of Interdisciplinary Health Sciences, Institute of Health and Society, Faculty of Medicine, University of Oslo, Oslo, Norway; 11 Department of Nursing Science, Institute of Health and Society, Faculty of Medicine, University of Oslo, Oslo, Norway; 12 Department for Cancer, Oslo University Hospital, Oslo, Norway

**Keywords:** Employment, Global health, Nationwide study, Sociodemographic factors

## Abstract

**Background:**

Prevalence studies are needed to assess the distribution of diseases. However, in a contrasting health promotion perspective, self-rated health is in itself an important field of study. This study investigated self-rated global health in the general population in Norway.

**Methods:**

As part of a national survey, a two-item measure of global health (score range 0–100) was administered to a general population sample, and 1776 of 4961 eligible participants (response rate 36%) responded. Group comparisons were conducted using independent *t*-tests and one-way analyses of variance, whereas factors associated with global health was investigated with linear regression analysis.

**Results:**

In the adjusted analyses, better global health was associated with higher age (*β* = 0.13, *p* <  0.001), having higher education (*β* = 0.10, *p* <  0.001), being employed (*β* = 0.21, *p* <  0.001), and living with a spouse or partner (*β* = 0.05, *p* <  0.05).

**Conclusions:**

While global health was similar for men and women in the Norwegian general population, other sociodemographic variables were linked with global health. In particular, the link between employment and self-rated global health was strong. The findings are considered representative for the Norwegian population.

## Introduction

The health status of a population is frequently estimated by prevalence of major diseases. Over the last decades, the wider impact of diseases has additionally been assessed by other measures, including disability adjusted life years (DALY) [1], which serves as an aggregated measure of disease burden. In a global perspective, the trend of decreasing DALYs related to communicable diseases and increasing DALYs related to non-communicable diseases was found to continue during the period 1990–2016 [2]. Data from the Global Burden of Diseases, Injuries and Risk Factors Study (GBD) were recently disaggregated to estimate the burden of disease in Norway in 2016 [3]. The findings reflected those of the global study: non-communicable diseases, like heart- and coronary disease; musculo-skeletal disease; cancer; dementia; and mental illness dominate the disease burden in Norway. This implies that Norwegian health services largely need to address the needs of persons living longer with disability [3], and consequently, the population’s health and quality of life in spite of disease will become increasingly important.

Knowledge about the distribution of diseases is needed; however, in a health promotion perspective, prevalence rates of diagnosable diseases provide limited information about the perceived health of a population. As established by the World Health Organisation (WHO) [4], health is a much broader concept than the mere absence of disease, encompassing the physical, mental and social well-being of a person. Further, as outlined by the International Classification of Functioning, Disability and Health (ICF) [5], health is a product of the ongoing interaction between the person, the environment and relevant disease conditions. Resulting from this interaction is the ability to function and cope with daily life, and to participate in desired activities and in society in general. In this perspective, health is not opposed to disease, but a positively defined state allowing the person to cope with life’s challenges and participate in society. In line with this view, Paterson [6] asserted that living with illness is living with wellness at the same time. Whether illness or wellness comes to the foreground of attention depends on several factors, such as time since diagnosis and symptomatic burden. However, it may also depend on towards which aspect – illness or wellness – the person and his or her surroundings direct their attention [7]. Similarly, studies in the field of mental health has argued the relevancy of conceptualizing health and illness as two separate, yet related concepts [8]. A Dutch study using a representative population sample found that those of higher age had less symptoms of mental illness, whereas their mental health was similar to that of younger participants [9]. This supports a view of health as conceptually different from the absence of illness.

Establishing health as an important concept in its own right, different from absence of disease, renders the challenge of measuring health and, in its extension, health-related quality of life. Well-known measures like the Short-Form Health Survey 36 (SF-36) [10] and the European Organisation for Research and Treatment of Cancer Quality of Life Questionnaire (EORTC QLQ-C30) [11] have been translated, validated and frequently used in a large number of countries worldwide, including Norway [12–15]. Although these are examples of well-performing instruments for assessing health-related quality of life, they are both extensive with 36 and 30 items, respectively. Thus, the two-item global health scale derived from the EORTC QLQ-C30 may be an alternative to measuring overall self-perceived health. In contrast to aggregated health measures, which are constituted from the person’s weighted response pertaining to each predetermined health dimension, global health measures takes into account the person’s own value system and interpretation of what matters most for his or her global well-being [16]. Previous Norwegian population studies have found higher global health among men compared to women [13, 15], and Hjermstad and co-workers also found higher global health among those with higher education and those with employment, compared to their counterparts [13].

In general, patient-reported outcome measures concerned with health and quality of life are needed because they provide information about the person’s own view [14], which may be quite different from the view of health professionals [17]. Although short scales can be criticized from a psychometric point of view [18], short and even single item measures of health have been found to have good validity [19]. They have the advantage of being flexible and easy to use [19, 20], and can be particularly fit to measure unidimensional outcomes emanating from multiple sources [21], such as global health. Previous Norwegian population studies that have used the global health scale are ageing [13, 15], and multivariate analyses of sociodemographic covariates to global health have not been performed. Therefore, more research is needed to assess global health in relation to the combined impact of a wider range of variables.

## Study aim

This study aimed to investigate differences in self-rated global health between groups and segments in the Norwegian general population, and to assess global health in relation to sociodemographic characteristics.

## Method

### Study purpose and design

The purpose of the Norwegian Population Study (NORPOP) was to gather data related to different self-evaluated health conditions and provide norm data for several questionnaires used for assessing symptoms, attitudes and behavior. The study had a cross-sectional survey design.

### Sample selection and size

We aimed at recruiting participants to constitute a representative sample of the Norwegian population [22]. The inclusion criteria were 1) ≥ 18 years of age, and 2) registered as a Norwegian citizen. The Central National Register selected a random sample where participants were proportionately stratified by age, gender and geographic region. Based on current knowledge concerned with response rates to mailed public health surveys [23], a response rate of 40% was estimated for the study. The sample size calculations, including the estimated response rate, indicated that a minimum of 5406 persons should be invited to participate. Therefore, during 2015 and 2016, the questionnaires were sent by regular mail to 5500 invited persons along with a letter explaining the study purpose. Of these, 1792 persons (36%) completed the questionnaires (32.6% of the targeted sample). Sixteen persons did not respond to the two questions included in the global health measure. After these had been excluded, the analyzed sample consisted of 1776 persons.

**Fig. 1:**
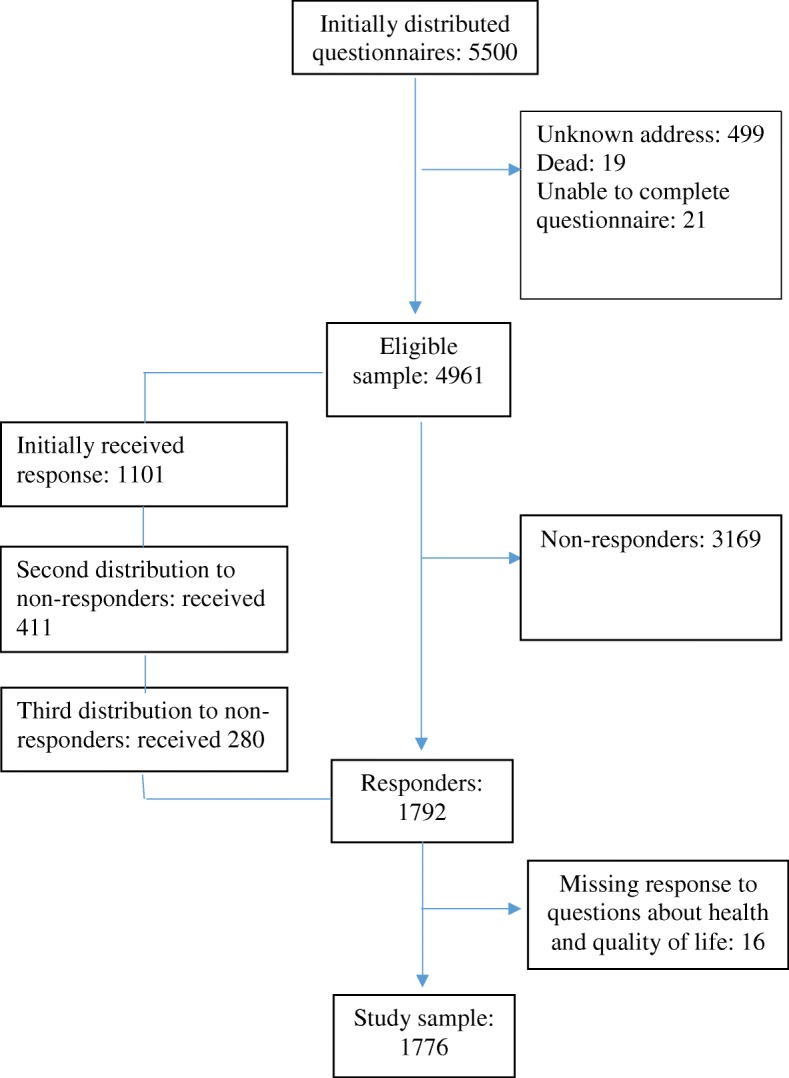
Flowchart showing the inclusion of the participants

The sample was compared with non-responders on the basic sociodemographic variables available. There were no significant differences between responders and non-responders with regard to mean age, gender or proportions living in rural and urban areas. A sample proportion of 66% were in paid work, compared to 67% in the general population [24]. A proportion of 17% lived alone in both groups. Still, in the sample 1.3% were without work and 53% had higher education, compared to 4.4 and 41.0% in the general population [22]. As a result, we consider our sample fairly representative of the general Norwegian population, although there was a larger proportion of the sample that had higher education. The flowchart in Fig. 1 shows the process of recruiting the participants to the study.

### Measures

#### Sociodemographic background

The collected sociodemographic data included age, gender, education, employment status, relationship status, and population size of place or city of residence. Age was categorized as 18–30 years, 31–40 years, 41–50 years, 51–60 years, 61–70 years, and 71 years of age or above. In the regression analysis, a continuous age variable was used. Educational level was categorized as 12 years or less (representing high school or less education) versus 13 years or more (representing some level of higher education). Employment was dichotomized as working versus not working, where the former category included being employed with paid work or undergoing education, while the latter category included full-time housework, being retired, unemployment or receiving disability benefits. In the regression analysis, relationship status was categorized as living with spouse/cohabitating versus not living with spouse/cohabitating. Population size of place or city was categorized as less than 2000 persons, 2000–19,999 persons, 20,000–99,999 persons, and 100,000 persons or more.

#### Self-rated global health

Self-rated global health was assessed with a measure based on two items from the EORTC QLQ-C30 [11]. The items were (i) “How has your health been during the last week?” and (ii) “How has your quality of life been during the last week?” The response format for both questions was an 11-point discrete scale anchored by the phrases “very poor” (0) in the lower end and “excellent” (10) in the upper end. The global health measure is established by calculating the average score for the two items and then transforming this score to represent a point on a 0–100 scale (i.e., multiply the raw score with 10).

### Statistical analyses

The sample of 1776 participants was included for analyses. Participants were excluded from analysis in the case of missing values on the relevant variables (casewise deletion). The data were analyzed using SPSS for Windows version 24 [25]. The sample distribution on global health was assessed with the Kolmogorov-Smirnov test. This variable deviated from the normal distribution (*p* <  0.001) and was skewed towards higher scores (*skewness* = − 1.00, *SE* = 0.06). However, deviation from the normal distribution with large samples such as this is commonly experienced and is not considered to compromise the validity of parametric statistical tests [26]. Moreover, non-parametric comparisons showed, with population size as the only exception, identical results as shown with the parametric tests. Therefore, we proceeded with the parametric analyses.

Differences in levels of global health status between groups were assessed by the independent *t*-test and one-way analysis of variance (ANOVA) as appropriate. Further, to assess relationships between the independent variables and self-rated global health, a multivariate regression analysis was conducted. Independent variables were included in one model (forced entry). The model included age, gender, education, employment status, relationship status, and population size of place of residence. Effect sizes (*ES*) were reported as standardized beta weights (*β*). The level of significance was set at *p* <  0.05 and all tests were two-tailed.

## Results

### Self-rated global health

The mean age of the participants was 53.2 years (*SD* = 16.6 years) and there was a higher proportion of females (53.1%) compared to males. The sample mean score on self-rated global health was 75.5 (*SD* = 21.2), and raw scores on health and quality of life were strongly correlated (*r* = 0.77, *p* <  0.001).

Table 1 displays the global health scores of the participants in sociodemographic groups and segments. Global health did not vary significantly by age group or gender. However, higher levels of education were significantly related to better global health (*p* <  0.001), with a large difference in mean score between those with the highest level (*M* = 79.4) versus the lowest level of education (*M* = 67.5). Employment status was significantly related to global health (*p* < 0.001), and inspection of the mean scores in the different employment categories revealed that persons who received disability pension had worse global health (*M* = 51.7) than all other employment groups (*M* ranging 68.3–75.8). Global health also showed an overall association with relationship status (*p* < 0.01) and population size (*p* < 0.05).

**Table 1:** Self-rated global health in sociodemographic groups

		Global health	
Characteristics	*n*	*M* (*SD*)	*p*
Age group	1758		0.96
18–30	209	75.3 (20.4)	
31–40	184	76.3 (18.9)	
41–50	352	75.1 (21.1)	
51–60	353	75.9 (20.7)	
61–70	395	75.5 (22.0)	
71 +	265	74.6 (22.6)	
Gender	1765		0.55
Men	828	75.8 (21.1)	
Women	937	75.2 (21.2)	
Education	1764		< 0.001
Elementary school, 7–10 years	138	67.5 (25.8)	
Secondary school or equivalent	491	73.1 (22.9)	
High school or equivalent	192	73.7 (21.8)	
College/university < 4 years	437	76.8 (19.2)	
College/university ≥4 years	506	79.4 (18.2)	
Employment	1760		< 0.001
In paid work	1073	78.0 (19.0)	
In education	90	75.8 (19.4)	
Retired	455	75.7 (22.0)	
Disability pension	109	51.7 (23.8)	
Housework/unemployed	33	68.3 (22.9)	
Relationships	1762		< 0.01
Spouse/partner	1271	76.6 (20.8)	
Unmarried/single	230	71.9 (23.0)	
Divorced/separated	97	72.1 (21.9)	
Widow/widower	75	72.4 (22.5)	
Steady relationship	89	75.1 (17.5)	
Population size	1754		< 0.05
Fewer than 2000	357	73.6 (22.7)	
2000–19.999	485	74.3 (22.0)	
20.000–99.999	421	77.3 (19.5)	
More than 100.000	491	76.4 (20.5)	

*Note. p* values indicate probability of differences between groups by *t-*tests (gender) or by one-way ANOVA (age group, education, employment, relationships and population size). Global health scores range 0–100, where higher scores indicate better global health

### Adjusted associations with self-rated global health

The results from the multivariate analyses are displayed in Table 2. Better global health was associated with higher age (*β* = 0.13, *p* < 0.001), having higher education (*β* = 0.10, *p* < 0.001), being employed or undergoing education (*β* = 0.21, *p* < 0.001), and living with a spouse or partner (*β* = 0.05, *p* < 0.05). The model was statistically significant (*F* = 15.4, *p* < 0.001).

**Table 2:** Linear regression analyses showing adjusted associations with self-rated global health (*n* = 1735)

Independent variables	Global health
Age	0.13**
Gender	−0.01
Education	0.10**
Employment	0.21**
Relationships	0.05*
Population size	0.03

*Note*. Table content is standardized beta weights (*β*), showing independent associations with self-rated global health. Variable coding: male (0), female (1); education < 13 year (1), education ≥13 years (2); without employment (0), employed (1); not living with spouse/partner (0), living with spouse/partner (1); higher values on age and population size are higher age and larger population, respectively

* *p* < 0.05, ** *p* < 0.01

## Discussion

This study of a Norwegian general population sample examined self-rated global health in sociodemographic groups and segments. Adjusted analyses showed that higher global health was significantly associated with higher age, having higher education, living in a paired relationship and, most notably, having employment.

The association between higher age and better global health seems counterintuitive at first glance. Higher age is normally associated with more disease and functional decline [13, 27], and therefore possibly associated with decreased global health – the latter concept also encompassing quality of life. As an example, the recent Norwegian population study using the SF-36 found that the mean scores decreased with age for all scales except for vitality, social functioning and mental health [14]. As the excepted scales are all subsumed under the mental health domain of the SF-36 [28], it appears that aging primarily affects physical health, and not mental health. Thus, the findings of the present study may be partly explained by our use of a relatively crude global health measure that does not specify particular aspects of health [11]. If older participants emphasized aspects related to mental health and quality of life when responding to the survey, they may have reported high global health in spite of declining physical health. The theoretical considerations of Paterson [6] and Keyes [8], essentially stating that health and illness are related, yet separate phenomena that do not determine each other, may also help to interpret the finding. Building on their views, the levels of global health among the older participants can be at a level comparable to those of the younger participants, in spite of potentially more diseases.

Having some level of higher education and having a spouse or cohabitant were associated with better global health. Conceptually, education can instill and foster the knowledge, attitudes and behaviors essential for maintaining good health, alternatively for addressing disease in a productive manner [7, 29]. Conversely, good health may also make education easier for people to take. Similarly, having a spouse or partner can provide the person with the social support needed to maintain or increase health, or to minimize the burdens of disease, thus contributing to maintain quality of life in spite of disease [30]. The findings of the present study are also consistent with those of previous population studies and clinical studies concerned with health-related quality of life. For example, in the latest survey using the SF-36 with a general population sample in Norway [14], a consistent linear pattern was shown, where those with more education had higher health-related quality of life in all domains compared to those with lower levels of education (all *p* < 0.001). With regard to the value of partnership, a study of persons with chronic obstructive pulmonary disease showed higher mental health scores among those who had a spouse or partner, compared to those who did not [31]. It should be noted, notwithstanding reaching statistical significance in our study, that the detected associations between global health and having higher education (*β* = 0.10) and having a spouse or partner (*β* = 0.05) were weak, according to the commonly employed criteria for assessing effect sizes [32].

The association between having employment and better global health, on the other hand, was of a moderate size (*β* = 0.21), and this is in line with a range of other studies. For example, Westerhof and Keyes [9] found that employment was associated with lower probability of mental illness, and a relationship between having employment and lower odds of experiencing current depression was recently found [33]. With a view to health in general, a study using data from the European Community Household Panel showed that the proportion of people in good health was substantially larger among those who reported to be employed, compared to their counterparts [34]. Due to the nature of our study, however, we cannot establish cause and effect associations. Possibly, employment can influence a person’s health, and vice versa: employment status, particularly in the long run, can be an effect of health status. In support of the latter, a Finnish study showed that becoming unemployed did not affect self-assessed health, whereas remaining unemployed in the long-term did [35].

Previous studies have suggested that observing mean differences of ≥10 points represent the minimum requirement for claiming a clinically relevant difference between groups [13, 15, 36]. A considerably larger difference was shown between those who were employed or in education, and those who were not. Given the political emphasis on sustaining high employment rates in the country [37], poorer health among those not employed can explain their position outside the labor market in the shorter or longer term. From another perspective, perceived stigma associated with being outside the workforce may be viewed as further decreasing the health among those who are not employed, as previously reported [38].

### Study strengths and limitations

The use of a large sample considered representative of the Norwegian population is a strength of this study, although we cannot rule out a selection bias related to participants’ willingness to participate in the study. The response rate was rather low, although similar to the response rate usually obtained in large population surveys [23]. Assessing concurrent predictors of global health in a multivariate analysis increases the trustworthiness of the results. Measuring global health using two items only can be viewed as a limitation. On the other hand, the advantage of such short measures lies in their feasibility. They are flexible, easy to administer, cost-efficient and have better face validity in comparison to multi-item scales [19]. Global health was conceptualized as a purely subjective phenomenon, and was measured as such. Thus, the results of the study do not speak about objective health or health as understood from an outsider’s point of view, but are exclusively concerned with the participants’ subjective reports.

## Conclusion

In this sample of the general population in Norway, self-rated global health was found to be associated with a range of sociodemographic variables, including age, education, employment and relationship status. The strongest link was shown between global health and employment, most likely representing a reciprocal association – those with poorer health are less likely to work, while employment may also contribute towards strengthening health. The study is important because it informs about global health perceptions from the person’s own perspective, as opposed to the outsider view often employed in health research. In addition, the identification of differences between sociodemographic groups and segments adds to the knowledge about the health of the Norwegian population in a comparative perspective. Moreover, it has allowed for an interpretation of the unequal importance of several sociodemographic factors associated with global health. The findings are considered representative for the Norwegian population.

## Data Availability

The datasets used and/or analysed during the current study are available from the corresponding author on reasonable request.

## Abbreviations

ANOVA: Analysis of variance
DALY: Disability adjusted life years
EORTC QLQ-C30: European Organisation for Research and Treatment of Cancer Quality of Life Questionnaire
GBD: Global Burden of Diseases, Injuries and Risk Factors Study
ICF: International Classification of Functioning, Disability and Health
NORPOP: The Norwegian Population Study
SF-36: Short-Form Health Survey 36
WHO: World Health Organisation
